# Disruption of Intracellular ATP Generation and Tight Junction Protein Expression during the Course of Brain Edema Induced by Subacute Poisoning of 1,2-Dichloroethane

**DOI:** 10.3389/fnins.2018.00012

**Published:** 2018-01-23

**Authors:** Gaoyang Wang, Yuan Yuan, Lanyue Gao, Xiaoqiong Tan, Guangqian Yang, Fenghong Zhao, Yaping Jin

**Affiliations:** ^1^Department of Occupational and Environmental Health, School of Public Health, China Medical University, Shenyang, China; ^2^Department of Health Laboratory Technology, School of Public Health, China Medical University, Shenyang, China

**Keywords:** 1, 2-dichloroethane poisoning, brain edema, ATP generation, blood brain barrier, tight junction associated proteins

## Abstract

The aim of this study was to explore changes in intracellular ATP generation and tight junction protein expression during the course of brain edema induced by subacute poisoning of 1,2-dichloroethane (1,2-DCE). Mice were exposed to 1.2 g/m^3^ 1,2-DCE for 3.5 h per day for 1, 2, or 3 days, namely group A, B, and C. Na^+^-K^+^-ATPase and Ca^2+^-ATPase activity, ATP and lactic acid content, intracellular free Ca^2+^ concentration and ZO-1 and occludin expression in the brain were measured. Results of present study disclosed that Ca^2+^-ATPase activities in group B and C, and Na^+^/K^+^-ATPase activity in group C decreased, whereas intracellular free Ca^2+^ concentrations in group B and C increased significantly compared with control. Moreover, ATP content decreased, whereas lactic acid content increased significantly in group C compared with control. On the other hand, expressions of ZO-1 and occludin at both the protein and gene levels in group B and C decreased significantly compared with control. In conclusion, findings from this study suggest that calcium overload and depressed expression of tight junction associated proteins, such as ZO-1 and occludin might play an important role in the early phase of brain edema formation induced by subacute poisoning of 1,2-DCE.

## Introduction

The compound 1,2-dichloroethane (1,2-DCE, CAS number: 107-06-2) is one of the most widely-produced halocarbons, used mainly in the production of vinyl chloride worldwide. This chemical is also used as general organic solvent, especially the thinner of adhesives in some countries. As a volatile organic chemical, 1,2-DCE evaporates quickly into the air, therefore, the primary route of exposure in the workplace is vapor inhalation (Liu et al., 2010; Sun et al., 2016c). It is known that subacute poisoning of 1,2-DCE can cause toxic encephalopathy in exposed workers. Postmortem examinations and clinical studies reported that brain edema was the main pathological change and cause of death among poisoned workers (Zhang et al., 2011; Chen et al., 2015). However, to date, little is known about the mechanisms of 1,2-DCE-induced brain edema.

Brain edema is commonly classified into cytotoxic and vasogenic types associated with either intracellular or extracellular accumulation of abnormal fluid. The former is due to disordered energy metabolism in injured brain cells, while the latter results from breakdown of the blood brain barrier (Michinaga and Koyama, 2015). It is well-known that intracellular energy metabolism is the process of ATP generation from the nutrients via oxidative phosphorylation in mitochondria. Both Na^+^-K^+^-ATPase and Ca^2+^-ATPase are the enzymes found in the plasma membrane of all animal cells, where they pump Na^+^ and Ca^2+^ out of cells while pumping K^+^ into cells against their concentration gradients, using energy provided by ATP hydrolysis. These transporters help maintain high intracellular concentrations of K^+^ and low concentrations of Ca^2+^ and Na^+^ (Jeremias et al., 2012; Liu et al., 2013). Disordered energy metabolism reduces ATP generation, and impedes activities of plasma membrane ion pumps, which will inevitably lead to excessive increase of intracellular Na^+^ and Ca^2+^, and finally cause water excess and calcium overload in brain cells, thus forming cytotoxic brain edema (Thrane et al., 2011; Song et al., 2014). It has been reported that recovery of Na^+^-K^+^-ATPase activity coincided with restoration of cerebral edema after brain hypoxia-ischemia (Mintorovitch et al., 1994). Our previous study (Sun et al., 2016b) also reported that exposure to 2-chloroethanol (a metabolite of 1,2-DCE *in vivo*) could cause decreased activities of Na^+^-K^+^-ATPase and Ca^2+^-ATPase in astrocytes, which might be related to 1,2-DCE-induced brain edema. Simultaneously, decreased ATP supply from mitochondria will promote anaerobic metabolism in the cytoplasm, leading to accumulation of lactic acid, and in turn, increased permeability of blood brain barrier (Kubota et al., 2012; Bosoi and Rose, 2014).

The blood brain barrier is a diffusion barrier essential for maintenance of normal brain function, which controls influx of most intravascular compounds from the blood into brain. It is composed of endothelial cells, pericytes and end-feet of astrocytes. Among them, endothelial cells are the most critical elements for preventing toxic substances from entering the brain. Endothelial cells are connected by tight junctions, which limit the paracellular flux of hydrophilic molecules across the barrier. Thus, tight junctions between endothelial cells are the most important structural components and crucial for permeability and integrity of the blood brain barrier (Wolburg et al., 2003; Abbott et al., 2010). Tight junctions consist of three integral membrane proteins: claudin, occluding, and junction adhesion molecules. In addition, there are several cytoplasmic accessory proteins including zona occuldens proteins (ZO-1, ZO-2, and ZO-3), cingulin and others. ZO-1 and occludin are thought to play the essential roles in maintaining the integrity of blood brain barrier, although there may be additional tight junction associated proteins (Jie et al., 2015). It has been reported that levels of ZO-1 and occludin were significantly altered in many pathological conditions, such as stroke, ischemia, hypoxia, septic encephalopathy, and brain tumors. In most cases, these pathological conditions were associated with breakdown of blood brain barrier and vasogenic cerebral edema (Hawkins et al., 2004; Liu et al., 2008; Jiao et al., 2011; Chen et al., 2012).

The results of our previous study (Wang et al., 2014) showed increased brain water content and enlargement of perinuclear and lacunar spaces surrounding vessels in the brain of mice exposed to 1.2 g/m^3^ 1,2-DCE for 3.5 h per day for up to three days, which suggested that a mouse model of brain edema induced by subacute poisoning of 1,2-DCE had been successfully established. Based on this model, the mechanisms underlying brain edema were explored in our previous study, and the results showed that protein expression of aquaporin 4 (AQP4) and mRNA expression of matrix metallopeptidase 9 in the brain were up-regulated in the early phase of brain edema formation. In the present study, alterations of cellular ATP generation and tight junction associated proteins, i.e., occludin and ZO-1, were further explored by using this mouse model of brain edema.

## Materials and methods

### Animals

Female Kunming albino mice weighing between 23 and 26 g were obtained from the animal laboratory of China Medical University. The animal room was kept at a temperature of 22–24°C with a 12 h light/dark cycle and a relative humidity of 50–60%. Mice were housed five per cage in the sterilized plastic cages with wood shaving bedding. Except when exposed to inhalants, food, and water were available to animals. During the study, mice were weighed daily and carefully observed for the signs of morbidity and mortality.

This study protocol has been approved by the Scientific Research Committee of China Medical University on Ethics in the Care and Use of Laboratory Animals, and was carried out in accordance with the National Institutes of Health guidelines in a manner that minimized animal suffering and animal numbers.

### Experimental procedures

After 1-week adaptation, 100 mice were randomly divided into four groups, a control group and three exposure groups, as described in our previous study (Wang et al., 2014). Mice in exposure groups were exposed to 1.2 g/m^3^ 1,2-DCE (initial concentration) for 3.5 h per day for one day (group A), two days (group B) or three days (group C).

### Treatment

Static inhalation exposure was used in this study. Mice from different groups were placed in the static exposure chamber with a capacity of 100 L, five in each chamber. Mice were exposed for 3.5 h per day for up to 1, 2, or 3 days. Solution of 1,2-DCE with purity of more than 99% was weighed according to administered concentrations, which were calculated by weight of 1,2-DCE divided by volume of chamber. The compound was placed on a filter paper in a plate suspended in the chamber, and then evaporated by a fan after sealing the chamber. Mice in the control group were kept in chamber without 1,2-DCE exposure. Following exposure, mice were removed from the chambers immediately.

Although it was problematic, static inhalation exposure has its own advantages, including that it is inexpensive, easy to build and operate, and involves less consumption of test chemicals. This is particularly suitable for small animals in experiments of acute and subacute inhalation exposure. Data from the U.S. Environmental Protection Agency (1998) and the Chinese Textbook of Toxicology, state that mice consume 1.38~2.22 L per hour of air, indicating that up to 10 mice were permitted in a 100 L exposure chamber for 4 h. However, in present study, only five mice were kept in 100 L exposure chamber for 3.5 h. At the end of exposure, the concentration of oxygen was near 20%, carbon dioxide was lower than 1.5%, and humidity was lower than 70% in the chamber. Moreover, the time-weighted average concentration of 1,2-DCE in the chamber during exposure was 0.99 g/m^3^.

### Analysis process

#### Activities of Na^+^-K^+^-ATPase and Ca^2+^-ATPase

Mice in each group were deeply anesthetized with ether and sacrificed by decapitation one day after the last exposure. The right cerebral hemispheres were removed immediately and homogenized in 0.9% cold physiological saline. Activities of both Na^+^-K^+^-ATPase and Ca^2+^-ATPase in cerebral homogenate were spectrophotometrically determined with commercially available kits (Nanjing Jiancheng Bioengineering Institute, China) according to manufacturer's instructions, and defined as the amount of inorganic phosphorus generated by 1 mg protein per hour (expressed as U/mg protein).

#### Contents of ATP and lactic acid

Cerebral homogenates were prepared as above and centrifuged at 12,000 rpm for 10 min. Supernatant was collected and analyzed spectrophotometrically by using the commercial assay kits (Nanjing Jiancheng Biotechnology Institute, China). The contents of ATP and lactic acid in the brain were expressed as μmol/g protein.

#### Concentrations of intracellular free Ca^2+^

The method described previously (Chan et al., 1996) was used in present study. Briefly, cerebral cortex was digested with 0.25% trypsin (Sigma, USA) at 37°C for 20 min. The reaction was terminated with DMEM containing 10% FBS (Hyclon, USA). Samples were filtered through a cell strainer, and centrifuged at 1,000 rpm for 5 min. The precipitated cells were resuspended in D-Hank's solution and adjusted to 1 × 10^6^ cells/ml. They were loaded with 5 μM fura-2 AM (Sigma, USA) at 37°Cfor 30 min in the dark, washed twice with D-Hank's solution, and incubated at 37°C for 5 min in the dark. Concentrations of intracellular free Ca^2+^ [Ca^2+^]i were determined by using fluorospectrophotometer (Hitachi F-4500, Japan). The following equation was used for calculation:

[Ca^2+^]i = Kd × [(R–Rmin)/(Rmax–R)] × (Sf380/Sb380), where Kd is the dissociation constant of the dye (224 nM was used); R is the ratio at excitation wavelengths 340/380 nm; Rmin is the ratio at zero [Ca^2+^]i, and Rmax is the ratio at saturated [Ca^2+^]i. Rmax was obtained by adding 0.2% Triton X-100 to make cell membrane permeable to Ca^2+^, allowing the extracellular and intracellular free Ca^2+^ to equilibrate. Thereafter, Rmin was determined by adding chelator to erase all extracellular and intracellular free Ca^2+^. Results were expressed as nmol/L (10^6^ cells/ml).

#### Immunofluorescence staining

Five mice in each group were anesthetized with ether. They were perfused through heart with PBS containing 0.02% heparin, followed by 4% paraformaldehyde in PBS. Cerebral cortexes were quickly removed to a cold plate, then fixed overnight in 4% paraformaldehyde. Fixed samples were immersed in 30% sucrose for 3 days and embedded in OCT-compound. Serial frozen coronal sections (8 μm) were sliced at −20°C using a cryostat microtome. Thereafter, sections were permeabilized for 30 min in PBS containing 0.3% Triton X-100 and incubated for 30 min with normal goat serum (ZSGB-BIO, Beijing, China) to block nonspecific binding of antiserum.

For immunofluorescence staining, sections were incubated with rabbit antibodies against ZO-1 (Millipore, CA, USA) and occludin (Zymed, MA, USA), and mouse antibody against GFAP (Millipore, CA, USA) at 4°C overnight. On the following day, goat anti-rabbit FITC and goat anti-mouse TRITC conjugated secondary antibodies (ZSGB-BIO, Beijing, China), were added and incubated for 30 min at 37°C in a dark room. Finally, the frontoparietal region was observed under a fluorescence microscope (Olympus BX50). Images were captured using a digital camera system (Olympus SC35). The relative fluorescence intensities of ZO-1 and occludin were quantified using image J software (NIH, USA).

#### Western blot analysis

Cerebral tissues were homogenized in RIPA lysis buffer, and then lysate was centrifuged at 4°C, 12,000 × g for 20 min. After centrifugation, supernatant was collected for SDS-PAGE. Protein concentrations were measured using BCA protein assay kits (Pierce, IL, USA). An equal amount of protein (45 μg per lane) was subjected to SDS-polyacrylamide gels and separated by electrophoresis. Subsequently, blots were transferred to polyvinylidene difluoride (PVDF) membranes (Millipore, MA, USA), and probed with rabbit antibodies against ZO-1, occludin or β-actin (Santa Cruz Biotech, Santa Cruz, CA, USA). Membranes were incubated with peroxidase conjugated secondary antibody. Immunoreactive bands were detected with an ECL kit (GE Healthcare, Buckinghamshire, UK). For quantification of immunoblot signals, band intensity was assessed semi-quantitatively by densitometry using an image analyzing software (Gel-Pro analyzer v4.0), and normalized by intensity of β-actin (as the internal control).

#### Quantitative real-time PCR assay

Total RNA was extracted from cerebral tissues using Trizol Reagent (Invitrogen, CA, USA). The first strand of cDNA was synthesized from total RNA using PrimeScript RT reagent Kits (Takara, Tokyo, Japan). Thereafter, cDNA was served as templates for real-time PCR amplification using SYBR Premix Ex Taq II (Takara, Tokyo, Japan) and ABI 7500 Real-Time PCR System (Applied Biosystems, CA, USA). To amplify a fragment of ZO-1, occludin and GAPDH (as house-keeping gene), the following primer pairs detailed in Table 1 were used. Amplification was conducted for 40 cycles of 5 s at 95°C and 34 s at 60°C. Results were analyzed using the comparative Ct method as described by Livak and Schmittgen (2001). RNA abundance was expressed as 2^−ΔΔCt^ for target gene normalized against GAPDH gene (as the internal control), and presented as fold-change vs. contralateral control samples.

**Table 1:** Oligonucleotide sequences used for real-time RT-PCR.

**Gene**	**Primer sequences**	**Product (bp)**
ZO-1	5′-AAGCGATTCAGCAGCAACAG−3′ 5′- GGACCGTGTAATGGCAGACT−3′	269
Occludin	5′- GCTATGGAGGCTATGGCTATGG−3′ 5′- CTAAGGAAGCGATGAAGCAGAAG−3′	161
GAPDH	5′- CAATGTGTCCGTCGTGGATCT−3′ 5′- GTCCTCAGTGTAGCCCAAGATG−3′	124

### Statistical analysis

Data were expressed as mean ± standard deviation (SD), and analyzed using SPSS for Windows, version 13.0 (SPSS Inc. IL, USA). Significant difference was evaluated by analysis of variance test (one-way ANOVA). *Post hoc* tests were analyzed by Student-Newman-Keuls test (SNK). Statistical significance was defined as *P* < 0.05.

## Results

### General health of mice affected by 1,2-DCE poisoning

As described in our previous paper (Wang et al., 2014), mice in group B showed body tremors and forelimb flexure. These signs were more severe in mice of group C. There were no abnormalities in control group during experimental period.

### Changes in Na^+^-K^+^-ATPase and Ca^2+^-ATPase activity, ATP and lactic acid content in the brain induced by 1,2-DCE poisoning

**Figure 1:**
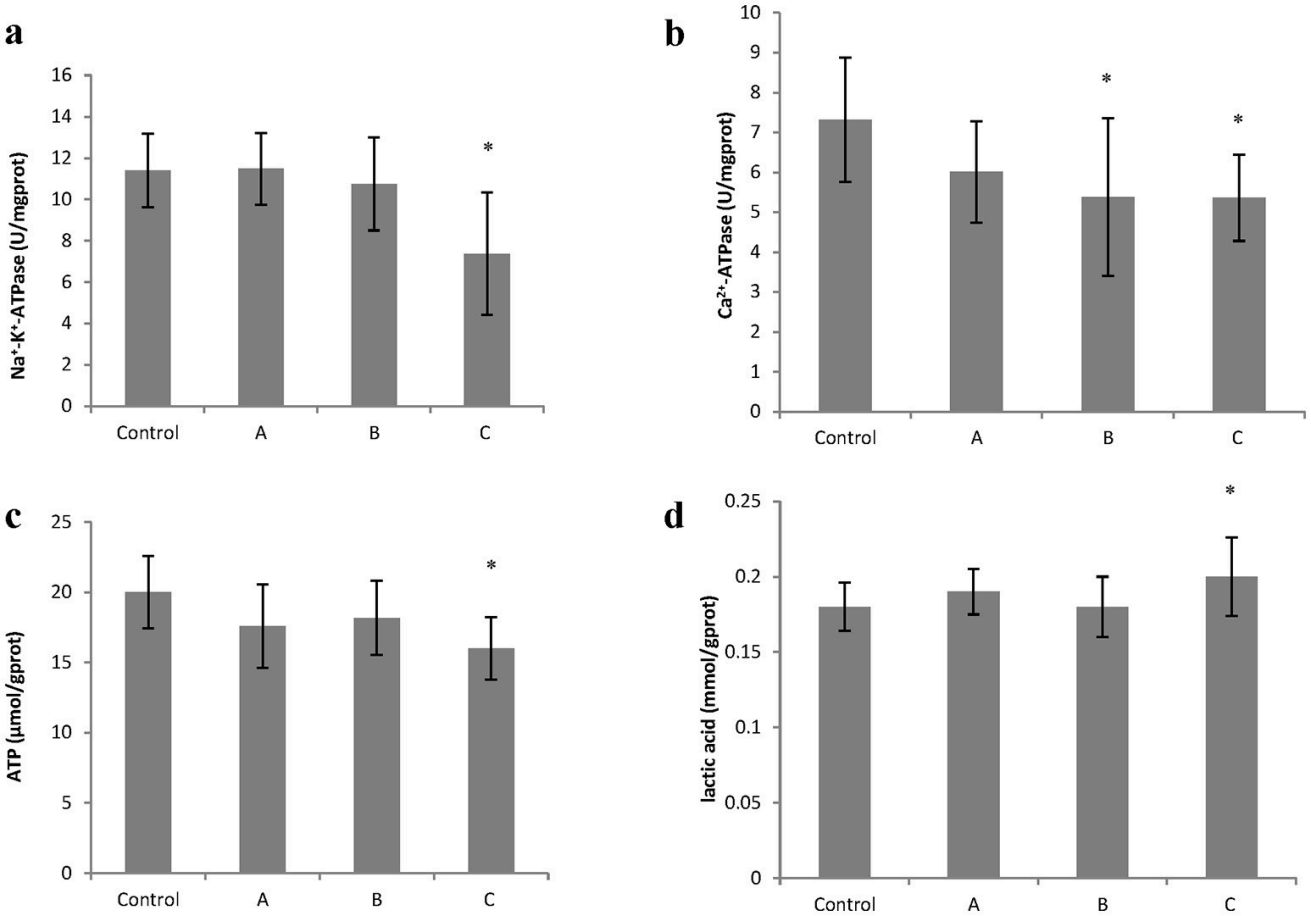
Changes in Na^+−^K^+^-ATPase and Ca^2+^-ATPase activity, ATP and lactic acid content in mouse brain cells induced by subacute poisoning of 1,2-DCE. **(a)** Comparison of Na^+^-K^+^-ATPase activity in mouse brain cells among groups; **(b)** Comparison of Ca^2+^-ATPase activity in mouse brain cells among groups; **(c)** Comparison of ATP content in mouse brain among groups. **(d)** Comparison of lactic acid level in mouse brain among groups. The number of mice were 10 in control group, 9 in group A, 8 in group B, and 8 in group C. Data were given as mean ± *SD*, and analyzed by One-way ANOVA. Significant difference was defined as *P* < 0.05. ^*^ vs. control group.

**Figure 2:**
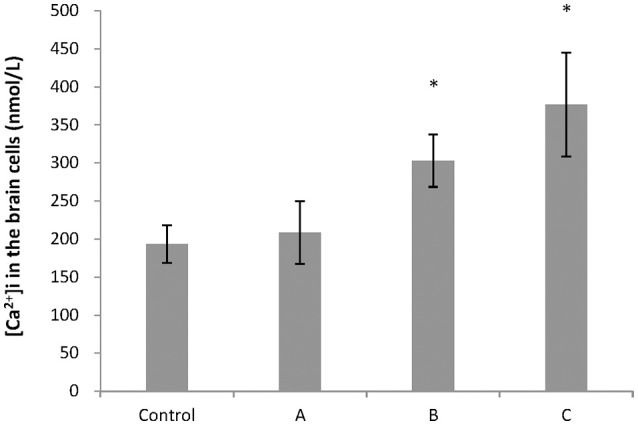
Comparison of intracellular free Ca^2+^ concentration in mouse brain cells among groups. Five mice in each group were selected for this analysis. [Ca^2+^]i represented concentrations of intracellular free Ca^2+^. Data were given as mean ± *SD*, and analyzed by One-way ANOVA. Significant difference was defined as *P* < 0.05. ^*^, vs. control group.

To explore changes in intracellular ATP generation in the brain of mice induced by 1,2-DCE poisoning, Na^+^-K^+^-ATPase and Ca^2+^-ATPase activities, ATP and lactic acid contents in mouse brain were determined. As shown in Figure 1, Na^+^-K^+^-ATPase activity in group C, and Ca^2+^-ATPase activities in group B and C decreased significantly (*P* < 0.05) compared with control. Moreover, compared with control, ATP content decreased significantly (*P* < 0.05), whereas lactic acid content increased significantly (*P* < 0.05) in group C. On the other hand, as shown in Figure 2, intracellular free Ca^2+^ concentrations in group B and C also increased significantly (*P* < 0.05) compared with control.

### Changes in ZO-1 and occludin expression in the brain induced by 1,2-DCE poisoning

**Figure 3:**
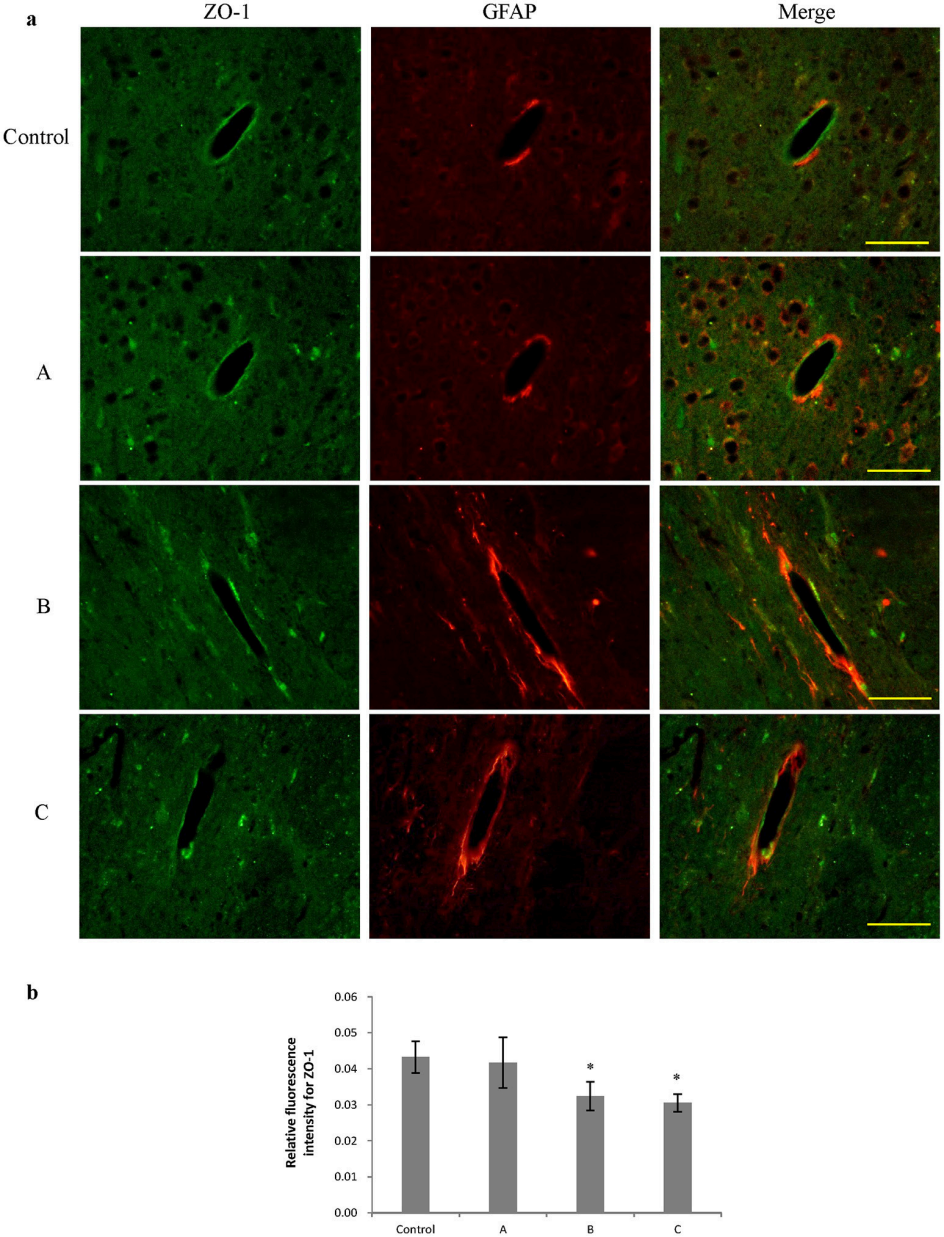
Comparison of ZO-1 protein expression in mouse brain among groups (scale bar = 25 μm). Five mice were selected in each group. Data were given as mean ± *SD*, and analyzed by one-way ANOVA. Significant difference was defined as *P* < 0.05, ^*^ vs. control group. **(a)** Immunofluorescence staining, green represented ZO-1, and red is for GFAP. A to C represented three exposure groups, in which mice were exposed to 1,2-DCE for 1, 2, or 3days. **(b)** Comparison of relative fluorescence intensity for ZO-1 among groups.

**Figure 4:**
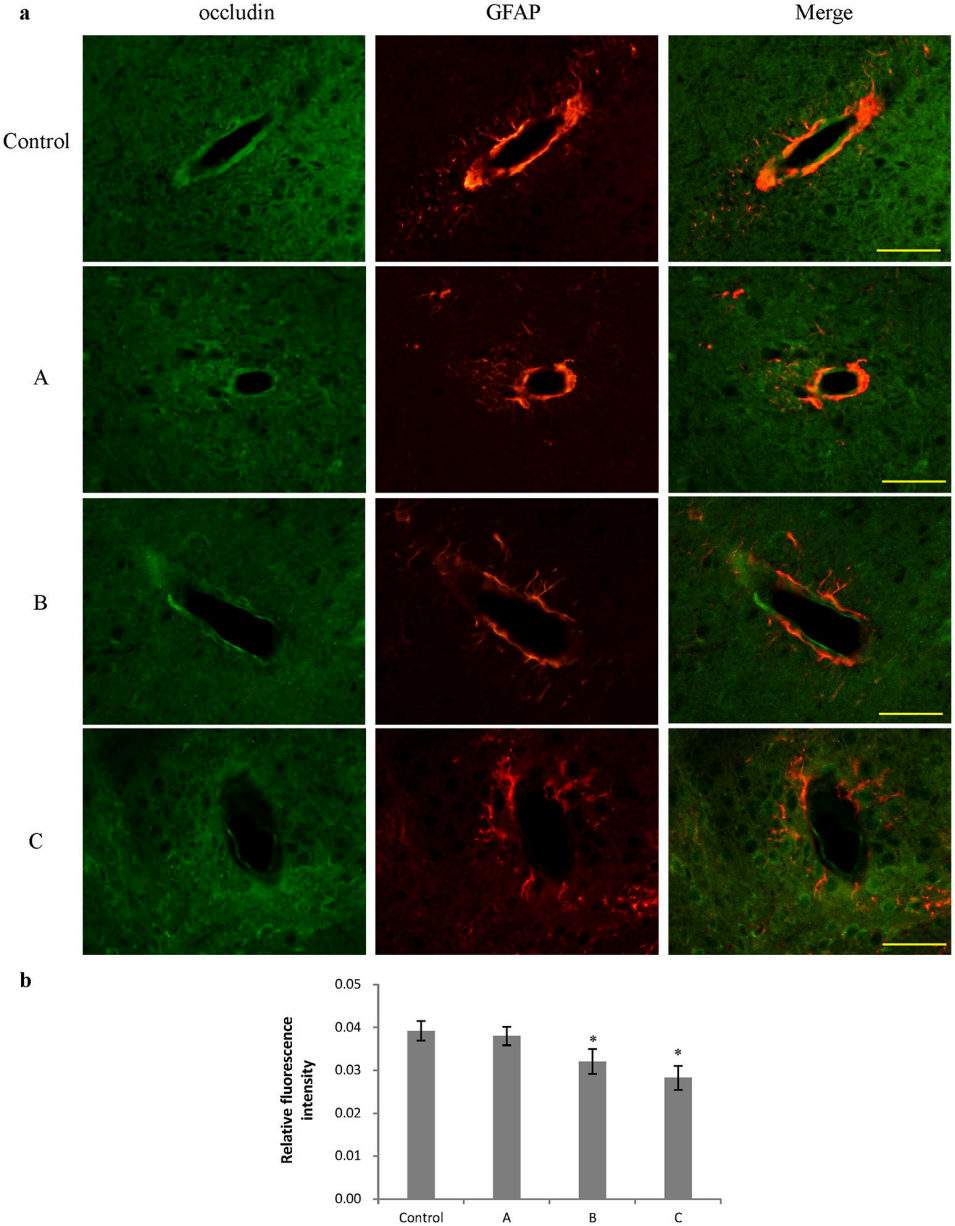
Comparison of occludin protein expression in mouse brain among groups (scale bar = 25 μm). Five mice were selected in each group. Data were given as mean ± *SD*, and analyzed by one-way ANOVA. Significant difference was defined as *P* < 0.05, ^*^ vs. control group. **(a)** Immunofluorescence staining, green represented occludin, and red is for GFAP. A to C represented three exposure groups, in which mice were exposed to 1,2-DCE for 1, 2, or 3 days. **(b)** Comparison of relative fluorescence intensity for occludin among groups.

Figures 3a, 4a show representative micrographs illustrating immunereactivity of ZO-1 and occludin in the brain, captured on different exposure days. There was continuous expression of ZO-1 and occludin in cerebral tissues in the control group, surrounded by GFAP-positive astroglia processes. However, expression of both ZO-1 and occludin in exposure groups, especially in group B and C appeared discontinuously. Furthermore, as shown in Figures 3b, 4b, compared with control, fluorescence intensities of ZO-1 and occludin in group B and C significantly decreased (*P* < 0.05).

**Figure 5:**
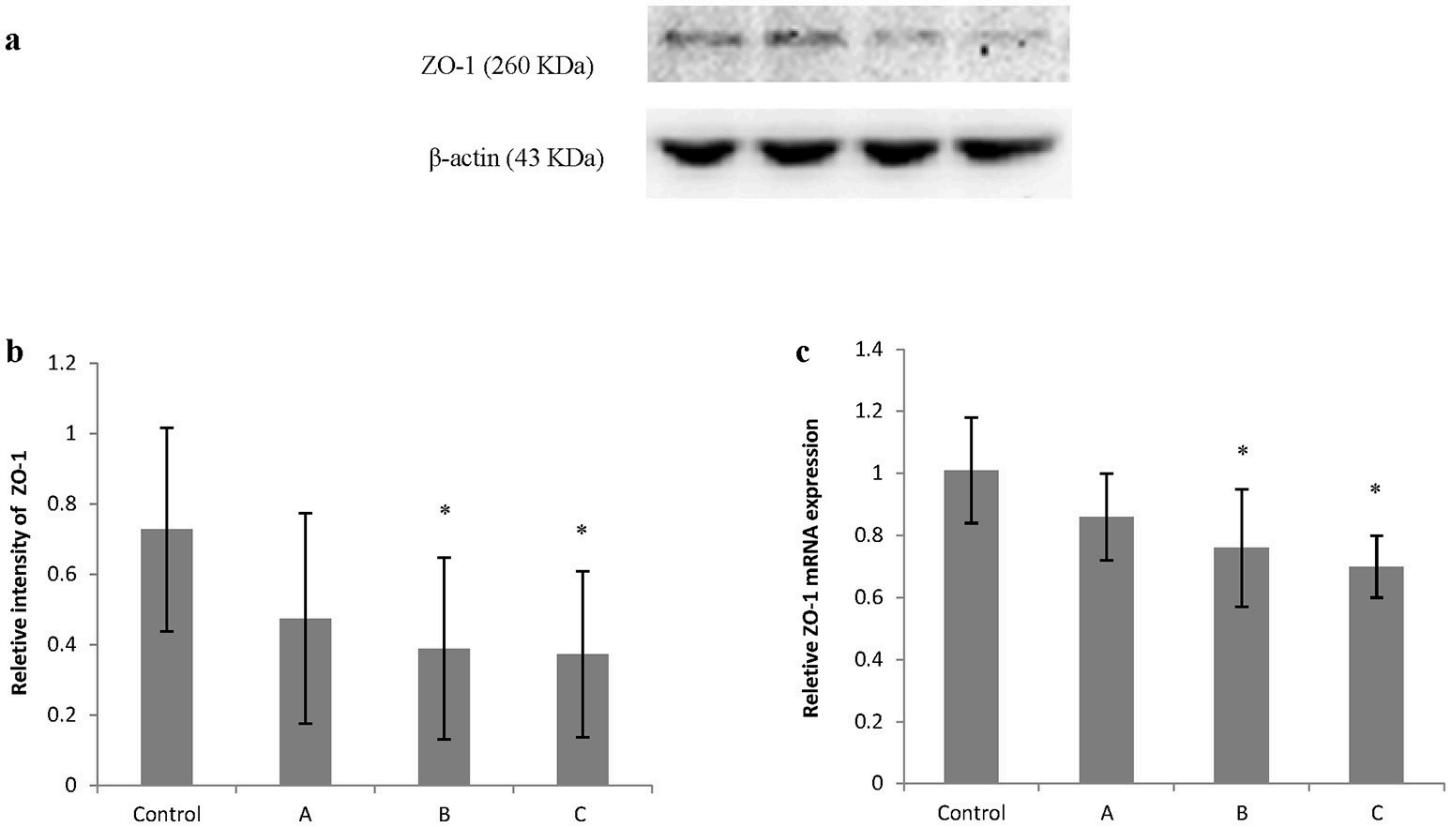
Comparison of ZO-1 protein and mRNA levels in mouse brain among groups. The number of mice used for Western blots and real-time RT-PCR were 10 in control, 9 in group A, 8 in group B, and 8 in group C. Data were given as mean ± *SD*, and analyzed by one-way ANOVA. Significant difference was defined as *P* < 0.05, ^*^ vs. control group. **(a)** Western blot analysis; **(b)** Densitometric analysis of Western blots; **(c)** Quantitation of mRNA by real-time RT-PCR. The mRNA levels were normalized to GAPDH and presented as fold change vs. control group.

**Figure 6:**
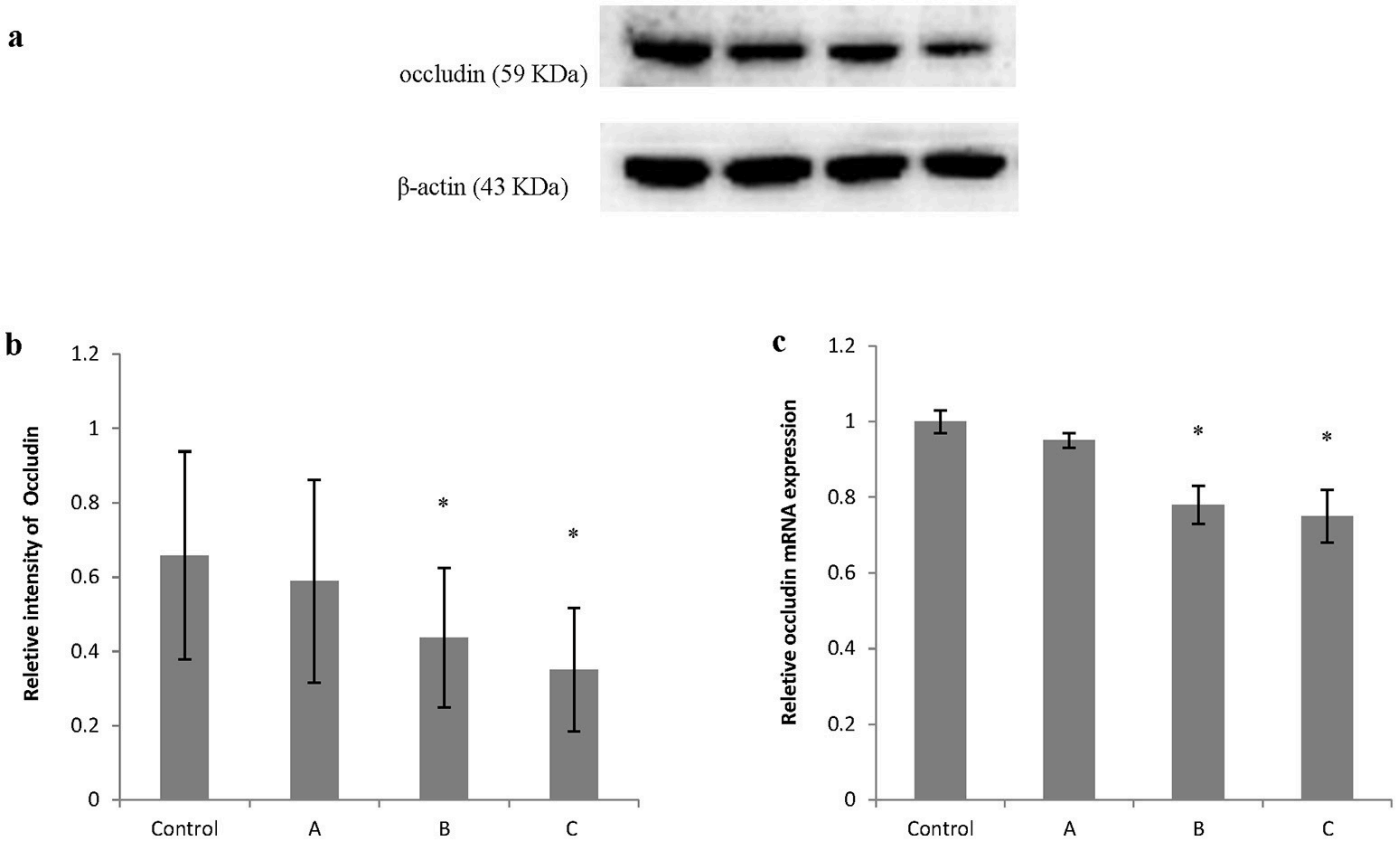
Comparison of occludin protein and mRNA levels in mouse brain among groups. The number of mice used for Western blots and real-time RT-PCR were 10 in control, 9 in group A, 8 in group B, and 8 in group C. Data were given as mean ± *SD*, and analyzed by one-way ANOVA. Significant difference was defined as *P* < 0.05, ^*^ vs. control group. **(a)** Western blot analysis; **(b)** Densitometric analysis of Western blots; **(c)** Quantitation of mRNA by real-time RT-PCR. The mRNA levels were normalized to GAPDH and presented as fold change vs. control group.

Figures 5a, 6a show typical western blots for ZO-1 and occludin. Figures 5b, 6b disclosed the quantitative analysis of protein blots. Consistent with the results of immunoreactivity, protein levels of both ZO-1 and occludin in group B and C decreased significantly (*P* < 0.05) compared with control. Additionally, graphs shown in Figures 5c, 6c disclosed quantitative analysis of mRNA expression, which demonstrated that mRNA levels of both ZO-1 and occludin in group B and C decreased significantly (*P* < 0.05) compared with control.

## Discussion

According to the results we reported previously (Wang et al., 2014), a mouse model of brain edema could be established by exposure to 1.2 g/m^3^ 1,2-DCE, 3.5 h per day for up to 3 days. The results in the present study showed decreased ATP levels and ATPase activities after 1,2-DCE exposure. On one hand, reduced ATP levels may be due to mitochondrial dysfunction or decreased oxygen supply induced by 1,2-DCE poisoning, which may contribute to decreased activities of these pumps since the energy derived from ATP hydrolysis is needed when the cells pump out Na^+^ and Ca^2+^ against their concentration gradients. On the other hand, decreased activities of the pumps may lead to excessive increase of intracellular Na^+^ and Ca^2+^, which may cause mitochondrial dysfunction and subsequently result in reduced ATP generation. In addition, the results in the present study suggested that suppressed Ca^2+^-ATPase activity might occur and lead to calcium overload in the early phase of 1,2-DCE-induced brain edema, since alterations of Ca^2+^-ATPase and intracellular free Ca^2+^ appeared earlier than other indicators after 1,2-DCE exposure. It is generally accepted that Ca^2+^-ATPase transports free Ca^2+^ into endoplasmic reticulum and out of the cell from cytoplasm using energy obtained through ATP hydrolysis. Thus, suppressed Ca^2+^-ATPase activity might result in excessive levels of cytoplasm free Ca^2+^, which could induce a series of catastrophic enzymatic processes, culminating in mitochondrial dysfunction (Putney, 2003) and certain brain pathologies (Paluzzi et al., 2007; Thrane et al., 2011; Song et al., 2014).

Therefore, our results suggest that exposure to 1,2-DCE might disturb calcium homeostasis in brain cells, thereby causing an energy metabolism disorder (Liu et al., 2013; Rosa et al., 2015; Vlodavsky et al., 2017). It has been reported by Wang et al. (2007) that treatment with 1,2-DCE could increase the concentration of calcium in rat neurons, which was consistent with our results. Similarly, in our previous study (Sun et al., 2016b), primary cultured astrocytes were exposed to different levels of 2-CE, and the result showed inhibited activities of Na^+^-K^+^-ATPase and Ca^2+^-ATPase in the cells, which is consistent with our present *in vivo* results.

Additionally, it is wellknown that lactic acid is the energy source for neurons in the brain. In injured neurons, less lactic acid may be consumed, leading to accumulation of lactic acid in the brain (Preuss, 2012; Bosoi and Rose, 2014). Nevertheless, intracellular contents of ATP and lactic acid are the important indicators of energy metabolism in the brain (Borutaite, 2010; Dienel, 2014). Though disordered energy metabolism is the main cause of cytotoxic edema in the brain, it might also result in vasogenic brain edema since increased levels of intracellular lactic acid induced by cytotoxic edema might disturb the function of the blood brain barrier (Chen et al., 2000; Rose, 2010; Yang et al., 2012; Afadlal et al., 2014). Therefore, although brain edema can be divided into cytotoxic and vasogenic edema at the initial stage, given that cytotoxic and vasogenic edema can be the cause of each other, it will become mixed brain edema soon.

Occludin, an integral protein at the tight junctions has four transmembrane domains, two extracellular loops and one intracellular loop (Fusco and Paluzzi, 1993). Among them, the extracellular loops originated from the neighboring cells form the paracellular barrier of tight junctions. The cytoplasmic domain is directly associated with ZO proteins for assembly of occludin into tight junctions. It has been reported that occludin is the most reliable immunohistochemical marker for tight junctions, reflecting structural integrity of blood brain barrier (Wells and Bonetta, 2005; Wen et al., 2014). ZO-1 belongs to the family of proteins known as membrane-associated guanylate kinase-like protein. It contains three PDZ domains, one SH3 domain and one guanyl kinase like domain. It has been reported that the guanylate kinase-like domain interacts with occludin, and the C-terminal binds to actin, forming a scaffold to anchor transmembrane proteins to the cytoskeleton in epithelial cells (Kojima et al., 2013). Based on the studies done so far, it is thought that both ZO-1 and occludin are essential for the integrity of the blood brain barrier (Jiao et al., 2011; Li et al., 2014; Mohamed Mokhtarudin and Payne, 2015).

Findings from this study demonstrated that both protein and gene levels of occludin and ZO-1 in the brain decreased apparently after two exposure days, suggesting that loss of tight junction associated proteins might occur at the early phase of 1,2-DCE- induced brain edema. In addition, our results suggest that expression of both ZO-1 and occludin were down-regulated at the transcriptional level in 1,2-DCE poisoned mice. Although the mechanisms concerning regulation of tight junction associated proteins are not completely understood, it is known that there are two principal signal transduction pathways: signals transduced from the cell interior to guide tight junction assembly, and signals transmitted from tight junctions to the cell interior to modulate gene expression. Multiple elements including Ca^2+^, protein kinase A and C, G protein and calmodulin, may be implicated in these processes. Among them, Ca^2+^ can act both intracellularly and extracellularly to regulate tight junctions. It has been reported that intracellular free Ca^2+^ plays a role in trans-endothelial resistance as well as in ZO-1 migration from intracellular sites to the plasma membrane (Brown and Davis, 2002). Saitou et al. (2000) reported that calcium deposits accumulated progressively in the cerebellum and basal ganglia in occludin -/- mice, and the small granular calcium deposits were often localized along small vessels, mainly venules and capillaries, suggesting that occludin was tightly associated with uptake and transport of free Ca^2+^. Extracellular calcium is also necessary for the homotypic interactions of E-cadherin, which is believed to be the initial event of junctional complex formation. When extracellular calcium is removed, there is a concurrent decrease in transmembrane electrical resistance and an increase in permeability (Huber et al., 2001). Therefore, in this study, it could be hypothesized that down-regulated gene and protein expression of both occludin and ZO-1 might contribute, at least in part, to depressed activity of Ca^2+^-ATPase, which could lead to enhanced concentration of intracellular free Ca^2+^. The accumulated results in our laboratory have demonstrated that up-regulated expression of MMP-9 and AQP4, and down-regulated expression of tight junction proteins, such as ZO-1 and occludin could be induced by 1,2-DCE poisoning during the course of brain edema. It is well known that up-regulated expression of MMP-9 could contribute to disruption of the blood brain barrier integrity. Moreover, findings from our studies have disclosed that activated Mitogen Activated Protein Kinase (MAPK) signal pathways were implicated in the modulation of proinflammatory factors, such as MMPs, iterleukins and tumor necrosis factor, AQPs, tight junction proteins during course of brain edema induced 1,2-DCE poisoning (Sun et al., 2016a; Wang et al., 2017).

Taken together, our data suggest that calcium overload and downregulated expression of tight junction associated proteins, such as occludin and ZO-1 might be the primary events occurring in the early phase of brain edema induced by subacute poisoning of 1,2-DCE. Accordingly, it was reasonable to speculate that brain edema might ensue from disordered intracellular calcium homeostasis and loss of tight junction associated proteins. Nevertheless, at present we could not address clearly how 1,2-DCE poisoning resulted in these alterations. Further studies are needed to provide us with clues to clarify the pathogenesis underlying 1,2-DCE-induced brain edema.

## Author contributions

GW: designed, performed, and interpreted the experiments and wrote the manuscript; YY: performed parts of biochemistry experiments and edited the manuscript; LG, XT, GY, and FZ: edited the manuscript; YJ: conceived the study, designed, and interpreted experiments and revised the manuscript. Authors listed in this paper participated in the design, execution, and analysis of this paper. All other authors have read the manuscript and have agreed to submit it in its current form for consideration for publication in the Journal.

### Conflict of interest statement

The authors declare that the research was conducted in the absence of any commercial or financial relationships that could be construed as a potential conflict of interest.
